# The effect of acute social isolation on neural molecular responses in components of the social decision-making network

**DOI:** 10.1186/s12864-024-10653-z

**Published:** 2024-08-08

**Authors:** Patricia C. Lopes, Madeleine Chang, Faith Holloway, Biola Fatusin, Sachin Patel, Chandler Siemonsma, Morgan Kindel

**Affiliations:** 1 Schmid College of Science and Technology, Chapman University, Orange, CA USA; 2 Neuroscience Graduate Group, University of Pennsylvania, Philadelphia, PA USA

**Keywords:** Social isolation, Acute, Chronic, Social decision-making network

## Abstract

Prolonged or chronic social isolation has pronounced effects on animals, ranging from altered stress responses, increased anxiety and aggressive behaviour, and even increased mortality. The effects of shorter periods of isolation are much less well researched; however, short periods of isolation are used routinely for testing animal behaviour and physiology. Here, we studied how a 3 h period of isolation from a cagemate affected neural gene expression in three brain regions that contain important components of the social decision-making network, the hypothalamus, the nucleus taeniae of the amygdala, and the bed nucleus of the stria terminalis, using a gregarious bird as a model (zebra finches). We found evidence suggestive of altered neural activity, synaptic transmission, metabolism, and even potentially pain perception, all of which could create cofounding effects on experimental tests that involve isolating animals. We recommend that the effects of short-term social isolation need to be better understood and propose alternatives to isolating animals for testing.

**Supplementary Information:**

The online version contains supplementary material available at 10.1186/s12864-024-10653-z.

## Introduction

Prolonged social isolation dramatically affects animals at various levels (molecular, physiological, and behavioural). For example, a study over the lifespan of singly or group-housed female rats, found isolation associated with dysregulated endocrine responses, increased anxiety and fearfulness, and increased malignancy of spontaneous mammary tumours [1]. Studies in *Drosophila melanogaster* found that, after social isolation, flies displayed altered aggressive, courtship, and sleep behaviour [2]. In humans, social isolation has been linked to increased likelihood of mortality [3]. Several other animal species have been shown to be affected by prolonged isolation [2, 4, 5].

Although the consequences of prolonged isolation are significant and influence health, reproduction, and survival, the impacts of brief isolation periods remain less understood. Short and prolonged isolation may not necessarily be expected to exert the same effects. For example, laboratory mice show a significant increase in aggression at 48 h after isolation, but not at 24 h [6]. A 30 min period of social isolation led to a ~ 2-fold increase in the expression of the neuropeptide tachykinin 2 gene in brain regions of mice, while a 2 week period of social isolation led to a ~ 6-fold increase [7]. Interestingly, several laboratory tests not aimed at investigating social isolation involve short periods of isolation. For instance, in respirometry studies, animals are placed alone in respirometry chambers for a few hours [8]. For several standardized rodent behavioural tests, animals may be placed alone in mazes or other behavioural apparatus for different short periods of time [9, 10]. In experiments where vocalizations are recorded, animals may also be isolated prior to or during the recordings for varying periods of time (e.g., 20 min prior to recording in [11]; 5 h 30 min prior to recording in [12]; and 24 h prior to recording, as well as isolation during recording [13]). How are these instances of acute isolation affecting the outcomes being quantified?

Here, we used a gregarious bird species, the zebra finch (*Taeniopygia guttata*), to test how an acute period (3 h) of social isolation affected neural molecular responses in brain regions that are part of the vertebrate social decision-making network [14, 15]. The social decision-making network consists of a collection of brain structures, comprised of the social behaviour network and the mesolimbic reward system, that regulates most forms of social behaviours and is evolutionarily conserved across vertebrates [14, 15]. Specifically, we focused on the hypothalamus (HYPO), the nucleus taeniae of the amygdala (TN) (a mammalian amygdala homologue), and the bed nucleus of the stria terminalis (BNST). In addition to regulating homeostatic functions, such as sleep, thermoregulation, and hunger [16–18], the hypothalamus contains several nuclei belonging to the social behaviour network, which are involved in aggressive, sexual, and parental behaviours [15]. The TN is the proposed equivalent to the mammalian medial amygdala (reviewed in [15]), and is involved in modulating behavioural responses to sexual and other social stimuli [19–22]. The BNST plays a role in aggressive and sexual behaviour [23–27], is involved in modulating stress and fear responses, and is proposed to assign valence to social stimuli [28–30]. More studies have been conducted on the effects of early life or long-term social isolation on the mesolimbic system, with structural alterations found in the amygdala, dopaminergic release and neural activation changes found in the ventral tegmental area, and transcriptional alterations in both (reviewed in [31]). Long-term social isolation also alters electrical responsiveness in the BNST and in various nuclei in the HYPO, following medial amygdala stimulation [32]. In addition, in the HYPO of prairie voles (*Microtus ochrogaster*), a decrease in oxytocin receptor expression was found after prolonged isolation, and a decrease in corticotropin-releasing hormone was found both under acute (1 h) and repeated social isolation [33, 34], suggesting that isolation can affect neuropeptide signalling in this region. We chose 3 h of social isolation because we had previously observed that acute changes to the social environment affected how finches responded to an immune challenge [35, 36]. In addition, this is a period that reflects the amount of time during which animals may be isolated for the types of tests mentioned previously (i.e., respirometry, behavioural phenotyping, and vocalization recording).

## Materials and methods

### Animals

Adult zebra finches purchased from Magnolia Bird Farm (Anaheim, CA) were maintained in indoor aviaries (2.1 m by 1.5 m by 2.1 m) at Chapman University for five weeks before the start of the experiments. Birds were housed in mixed sex groups of 18 animals and provided with food (German millet mixed with canary seed), cuttlebones and water *ad libitum*. No nesting cups or nesting materials were provided. The light/darkness schedule was 13 L:11D, with lights on at 06:30 in the morning.

### Treatments

On the day prior to the experiment, pairs of animals (one male and one female from the same aviary) were moved together to an experimental cage (41.9 cm by 30 cm by 55.9 cm) located in a separate testing room. The cage contained two perches, one food cup and one drinking bottle. Experiments took place in the morning. At 8:00, males received an intra-muscular injection of sterile phosphate buffered saline (Endotoxin-free PBS, Sigma-Aldrich TMS-012-A) as part of a separate experiment [36]. After the injection, the female in each cage was captured and either immediately placed back in the respective cage or permanently removed (and placed back in their original aviary). This procedure therefore created a treatment where males remained paired with a female (Paired treatment, *n* = 6) or where they became isolated (Isolation treatment, *n* = 11). Three hours later, males were euthanized via isoflurane inhalation, followed by decapitation. Brains were immediately extracted and flash frozen. Serum was collected from centrifuged trunk blood. These tissues were obtained within 3 min of entering the experimental room. Brains and serum were preserved at -80 °C until processing.

### Brain region dissection

Brains were coronally sectioned at -18 °C, using a Leica CM1860UV cryostat. The brain regions of interest, namely the hypothalamus (HYPO), the nucleus taeniae (TN) and the bed nucleus of the stria terminalis (BNST), were identified based on the zebra finch brain atlas [37]. Each region was collected using distinct surgical micropunches (EMS Rapid Core Instruments) and punches were obtained from 100 μm slices, spaced apart by three 30 μm slices (Fig. S1). The internal diameter of the micropunch tools was 4 mm for HYPO and 3 mm for TN and BNST. Brain tissue from each region was preserved in separate beaded lysis tubes (ZR BashingBeads Lysis Tubes 2 mm, Zymo Research, item S6003-50), containing 1 mL of QIAzol lysis reagent (Qiagen, item #79,306). The beaded tubes containing the brain tissue were agitated at 7 m s^− 1^ for 20 s on a Beadbug 6 homogenizer (Benchmark Scientific) and then allowed to rest for 5 min. The homogenate was transferred to a new tube and kept at − 80 °C until the RNA isolation procedure.

### RNA isolation

RNA was isolated from the aqueous layer produced after chloroform precipitation, using the RNA Clean & Concentrator Kit-5 (Zymo Research, item # R1013) following manufacturer’s instructions and including the DNase I in-column treatment step. For the Paired treatment, RNA of sufficient quality was recovered from 5 out of the 6 animals. For the Isolated treatment, RNA was only extracted from a randomly selected set of samples (from 6 animals), all of which yielded sufficient quantity and quality. These 11 RNA samples were sent to Novogene Corporation Inc. (Chula Vista, CA, USA) for quantification, library preparation, and sequencing.

### RNA sequencing and quantification

RNA quantification, integrity, and purity were obtained on an Agilent 2100 Bioanalyzer (Agilent Technologies, Santa Clara, CA). The NEBNext^®^ Ultra™ RNA Library Prep Kit for Illumina^®^ (NEB, USA) was used to prepare cDNA libraries and the cDNA fragments (150 ~ 200 bp in length) obtained were then purified with the AMPure XP System (Beckman Coulter, Beverly, USA). Paired-end sequencing of libraries (PE150; Illumina Novaseq 6000) was performed using standard protocols. An average of 48.3 million paired-end raw reads per sample were obtained. An average of 75.91% of clean reads (i.e., after adapter removal and quality filtering) were mapped to the reference genome (taeGut3.2.4, release 96, downloaded from Ensembl) using HISAT2 [38]. HTSeq (v0.6.1) [39] was used to count numbers of mapped reads. Mapping statistics are in Table S1.

### Bioinformatic analysis

#### Differential gene expression analysis

Differential gene expression analysis was performed using DESeq2 package (v.1.42.0) [40], to study the effect of the social treatment on gene expression. A principal component analysis (PCA) of normalized and variance stabilized counts was applied to the entire dataset using the plotPCA function from the package BiocGenerics v. 0.48.1, demonstrating clear separation of the samples along PC1 and PC2 based on brain region (Fig. S2). For each brain region, we then filtered low count genes as described in the pre-filtering step of the DESeq2 vignette. In this way, genes with less than 10 raw counts total across at least 5 samples were filtered, which resulted in 13,518 genes for HYPO, 13,569 for TN, and 13,533 for BNST left for analysis. After filtering, a PCA was applied to each brain region, revealing one outlier sample in HYPO which was separated from all other samples on both PCA axes (Fig. S3). This sample (HY5, from the Isolated treatment) was therefore removed, resulting in a HYPO sample size of 5 Isolated and 5 Paired animals, and containing 13,443 genes after repeating the pre-filtering step. Each brain region was analysed separately, and gene counts were modelled as a function of social treatment. The p-values resulting from Wald tests were adjusted using the Benjamini–Hochberg procedure to control for false discovery rate due to multiple testing. When these adjusted p-values (henceforth *p*_adj_) were < 0.05, genes were considered significantly differentially expressed between treatments.

#### Gene set enrichment analysis (GSEA)

To understand how different biological pathways were affected by acute isolation, we performed gene set enrichment analysis. This was done using the GSEA function in the clusterProfiler package (v. 4.10.0) [41], with a minimum set size of 10 genes and maximum set size of 500 (defaults), and using FDR as the adjustment method for multiple comparisons, with an adjusted p-value cutoff of < 0.05. Ranked lists of genes used as input in the GSEA analysis were obtained by multiplying the sign of fold change (i.e., the direction of change) by the negative log_10_ p-value obtained from the DESeq2 analysis, following [42]. These scores were then rank ordered by decreasing value. We focused here on gene sets relevant to the brain by creating a list of GO terms that combines the GO terms found in the SynGO [43] and the NIGO [44] lists of Gene Ontologies. Using that list, a separate analysis was done for each Gene Ontology collection (i.e., Biological Process, Cellular Component, and Molecular Function). Dotplots for enrichment results were also prepared using the clusterProfiler package.

#### Behaviour

The GSEA results led us to examine videorecorded behaviours of the 11 sequenced animals. Behaviour was recorded over the 1 h period immediately preceding euthanasia using Axis M1065L network cameras (Axis Communications). Behaviour was analysed over a 50 min period, with a 10 min gap in the middle. The gap corresponded to the 5 min before and after a researcher was in the room. We focused on analysis of time resting [defined as amount of time (in seconds) spent immobile (i.e., not performing the other target behaviours, nor drinking, preening, or vocalizing)], number of hops, and number of times pecking at the inside of the food cups (coded as eating). The residuals of resting were normally distributed, and resting was analysed by using a Welch’s t-test. The other two behaviours consisted of count data and were analysed using a non-parametric test, the Kruskal-Wallis rank sum test. Results were considered statistically significant if p-value < 0.05.

### Corticosterone

Corticosterone was quantified from serum samples using an enzyme immunoassay kit (ADI-900-097, Enzo Life Sciences, Ann Arbor, MI) in single microplate and calculated as described in [36]. Sufficient serum was available from 4 Isolated animals and 5 Paired animals, but not all these samples come from the animals for which the brains were sequenced (Table S6). Corticosterone was not normally distributed due to an outlier sample and was boxcox transformed for analysis. Analysis was done using a Welch’s t-test and a p-value < 0.05 was considered statistically significant.

### Statistical analyses

All statistical analyses in this study were carried out in R v. 4.3.2 [45], and unless otherwise noted, plots were prepared using the ggplot2 package (v. 3.4.4) [46]. Boxplots represent the median, the first and third quartiles (lower and upper hinges), and the smallest, and largest values (lower and upper whiskers) no further than the interquartile range.

## Results

### Genes differentially expressed under acute social isolation

**Fig. 1:**
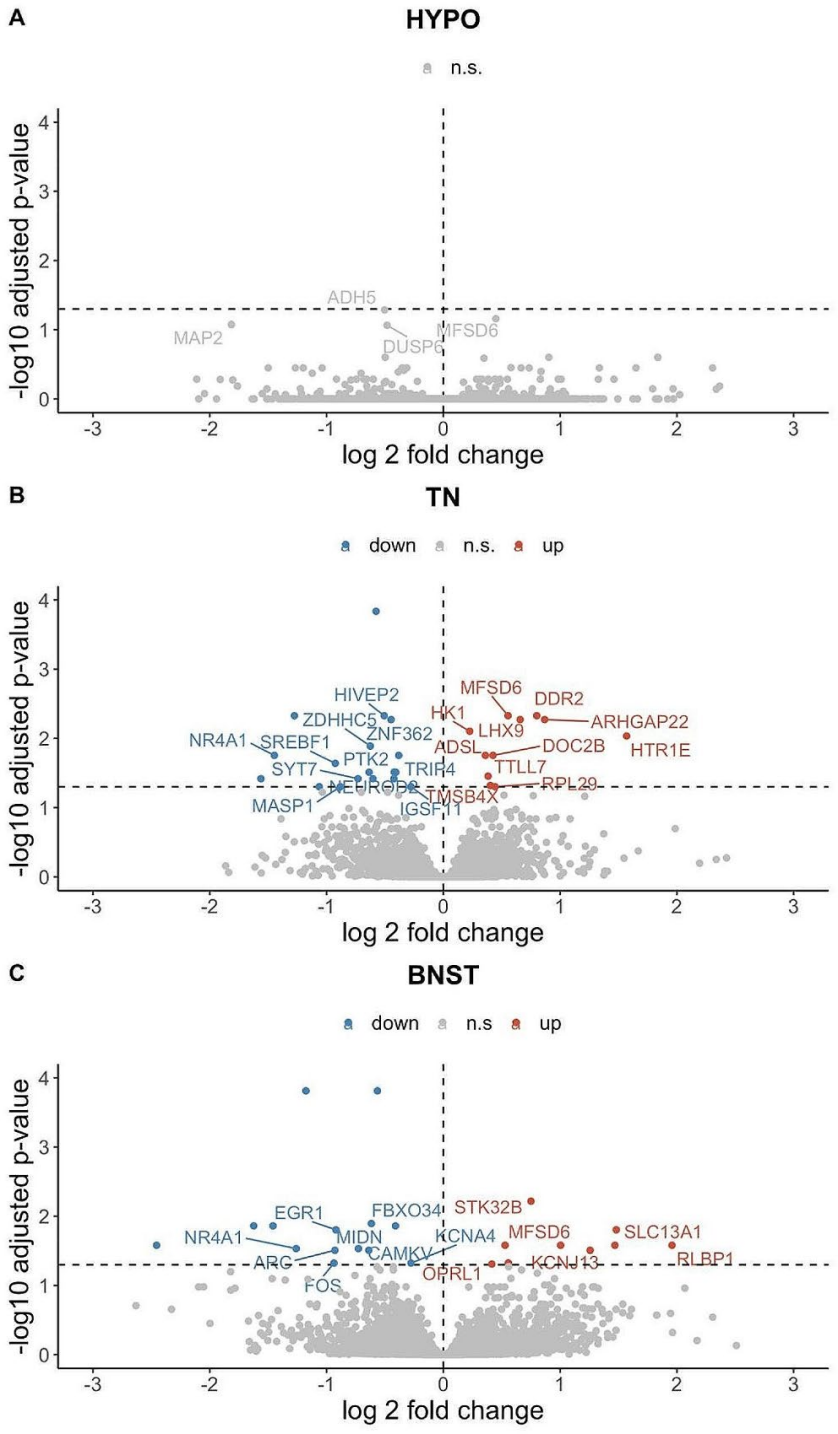
Differentially expressed genes in response to acute isolation in the hypothalamus (**A**), nucleus taeniae (**B**), and the bed nucleus of the stria terminalis (**C**). Genes with p-adjust < 0.05 without a symbol do not have a HUGO Gene Nomenclature Committee (HGNC) Symbol

Animals that spent 3 h in isolation (Isolated) had zero differentially expressed genes (DEGs) relative to Paired animals in the hypothalamus (HYPO), 29 in the nucleus taeniae (TN), and 24 in the bed nucleus of the stria terminalis (BNST) (Tables S2, S3 and S4, respectively; Fig. 1). Only one gene was upregulated after isolation across the three brain regions: *MFSD6* (at a *p*_adj_ <0.1 in HYPO; Fig. 1). In terms of additional overlaps between brain regions, in both the TN and BNST *NR4A1* and ENSTGUG00000018608 (a microRNA) were downregulated (at *p*_adj_ <0.05), with *NR4A1* being one of the genes with the largest negative fold change in both regions. If considering a slightly more relaxed *p*_adj_ cutoff, *MIDN*, a DEG in BNST (*p*_adj_ <0.06) was also downregulated in TN, and *TRIP4*, a DEG in TN was also downregulated in BNST (*p*_adj_ <0.06). Two additional genes of interest to highlight are the only DE neuropeptide receptors: *HTR1E*, a serotonin receptor that was upregulated in the TN, and *OPRL1*, an opioid receptor that was upregulated in the BNST.

Since a study of overnight isolation has been carried out in female zebra finches [47], we examined whether DEGs with the greatest effect size in that study could also be found in our study. Even though that study was conducted on a different brain region, the auditory lobule, we found 5 genes that were downregulated in both studies: *FOS*, *ARC*, *EGR1*, and *MIDN* (all in the BNST) and *HIVEP2* (in the TN).

### Pathways influenced by acute social isolation

**Fig. 2:**
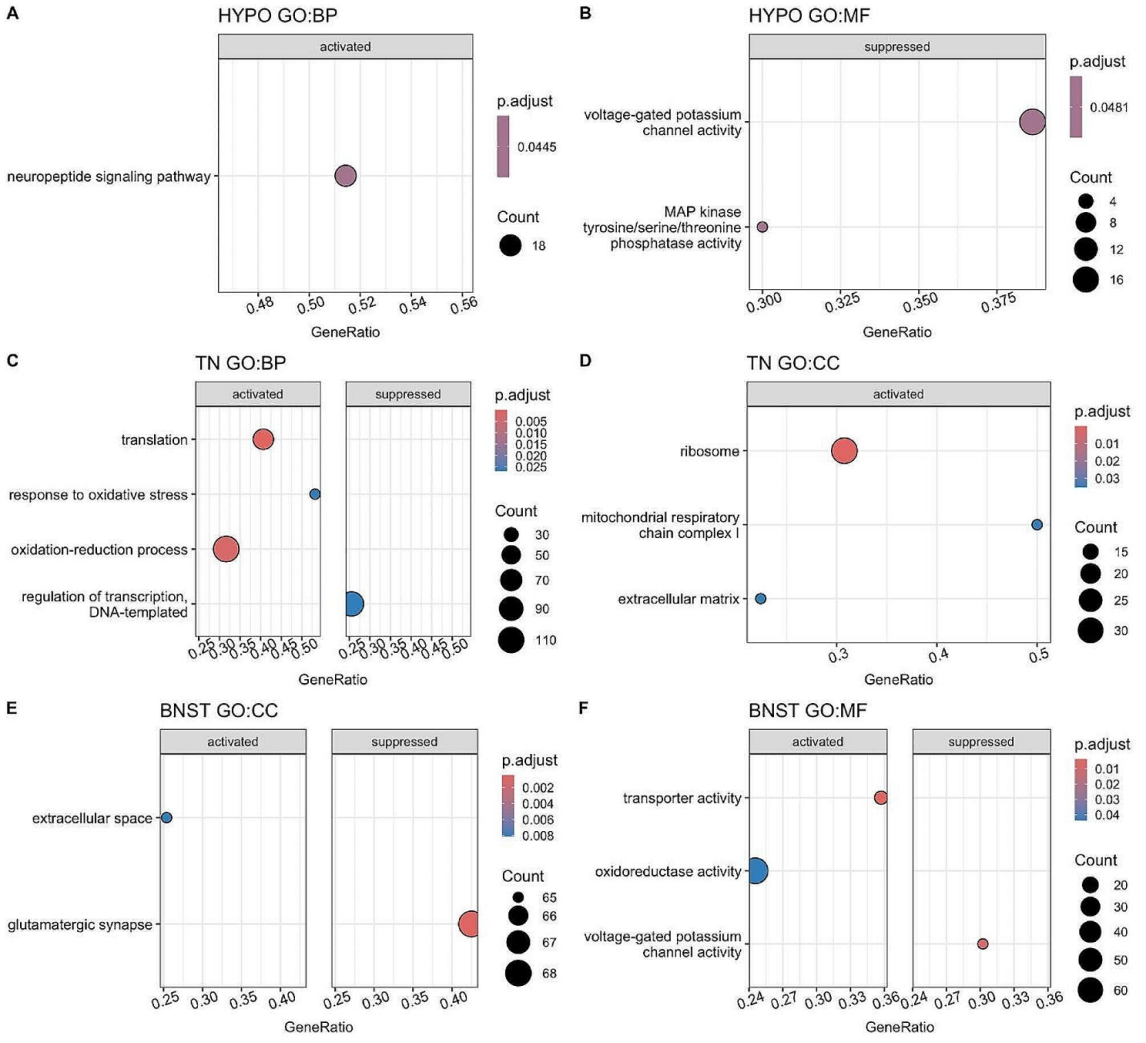
Gene Set Enrichment Analysis results. Results show all enriched pathways detected within each GO category, for each brain region. The absence of a GO category plot indicates that no pathway was detected for that category. The size of the circles corresponds to the number of genes that responded to the Isolation treatment in a given pathway

In addition to determining the genes that most drastically changed in expression after acute social isolation, we also studied whether classes of genes that responded to the social treatment were over-represented in any brain region studied (Fig. 2, Table S5). Pathways containing upregulated genes were related to neuropeptide signalling in HYPO, translation in the TN, and oxidation-reduction in both the TN and the BNST. Downregulated pathways were related to voltage-gated potassium channel activity in both the HYPO and the BNST, regulation of transcription in the TN, and glutamatergic synapse in the BNST.

### Behaviour and corticosterone

**Fig. 3:**
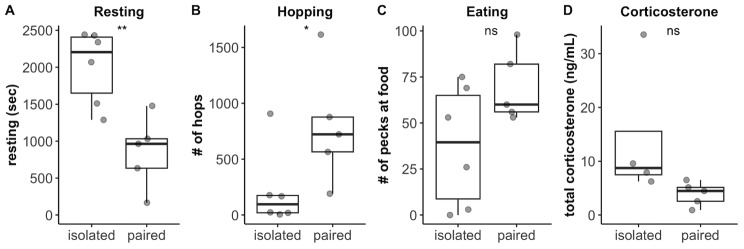
Behaviours (**A**-**C**) (isolated: *n* = 6; paired: *n* = 5) and circulating corticosterone (**D**) (isolated: *n* = 4; paired: *n* = 5) of animals in Isolated relative to Paired treatment. ** used for p < = 0.01, * used for p < = 0.05, ns used for *p* > 0.05

Because hypothalamic neuropeptide signalling is critically important for the regulation of numerous physiological and behavioural processes [48, 49], we investigated the genes contained in the only pathway activated in the HYPO. Out of the 18 genes in the neuropeptide signalling pathway gene set identified by GSEA, 4 are involved in regulating feeding behaviour: *HCRTR2*, *NPY1R*, *AGRP*, and *NMUR2* [50]. For this reason, we examined video-recorded data to assess whether treatment was associated with differences in behaviour, particularly feeding behaviour (Fig. 3). While Isolated animals were significantly less active then their Paired counterparts, shown by increased resting time (Welch Two Sample t-test: t = 3.8786, d.f. = 8.7153, p-value = 0.003975) and decreased hopping (Kruskal-Wallis test: χ^2^ = 4.0333, d.f. = 1, p-value = 0.04461), feeding behaviour was not significantly different between treatments (Kruskal-Wallis test: χ^2^ = 2.4193, d.f. = 1, p-value = 0.1198). There was, however, larger variation in feeding behaviour in Isolated than in Paired animals (Fig. 3C).

Isolation can increase circulating corticosterone in zebra finches relative to group housing (e.g., [47, 51, 52]). Since glucocorticoids are pleiotropic hormones, capable of affecting several aspects of physiology and behaviour [53], we compared free circulating levels of corticosterone between treatments at the time of brain collection. While overall, corticosterone appeared to be elevated in the Isolated animals, this difference was not statistically significant (Welch Two Sample t-test: t = 2.4053, d.f. = 6.3231, p-value = 0.05082; Fig. 3D).

## Discussion

While previous research has shown that prolonged social isolation affects animal physiology, less is known about short term isolation. The current study was aimed at understanding whether a short period of isolation can change neural gene expression in zebra finches. We found large changes in expression in 29 or less genes per brain region studied. In addition, we found coordinated changes in gene expression in pathways indicative of alterations in synaptic transmission, neuropeptide signalling, and metabolism. These results suggest that even short periods of isolation kickstart neural changes that could lead to confounding results in experiments where separation from groupmates is necessary but not the goal of the study.

The only gene that was upregulated after isolation across all brain regions studied (albeit not significantly so in HYPO at p-adjust < 0.05) was *MFSD6*. MFSD6 is an atypical solute carrier (SLC) of the major facilitator superfamily. Like several other atypical SLCs (reviewed in [54]), the expression of *MFSD6* is altered by nutritional status and food intake in the brains of rodents [55] and in *Drosophila melanogaster* [56]. In our experiment, we cannot directly link the upregulation in *MFSD6* to altered food intake, since at the time point analysed, we detected no significant differences in food intake between treatments. We also did not quantify differences in food intake prior to the social manipulation, which could have led to variation in hunger at the time point analysed. It is also possible that Isolated animals fed less at an earlier time point during isolation, thereby activating hunger signals, or that they would have fed differently at a later time point after the shift in gene expression. In fact, 4 of the neuropeptides in the only pathway activated in the hypothalamus are involved in the modulation of appetite and food intake [50]. An experiment in humans using functional magnetic resonance imaging to quantify neural responses suggested that 10 h of social isolation (acute isolation) evoked craving responses in the midbrain that are similar to the ones cause by hunger after fasting [57]. The brains of *Drosophila melanogaster* after chronic isolation show activation of hunger signals and resemble, from a transcriptomic perspective, the brains of flies after 24 h of starvation [58]. It is therefore possible that social isolation affects patterns of food intake, hunger, or interest in food, which would be an important consideration in tests for food preferences or for drive to eat in isolated animals.

The other gene showing differential expression between the treatments in at least two brain regions was *NR4A1*, which was downregulated in Isolated animals in both the TN and the BNST. NR4A1 is an immediate early response gene and is rapidly expressed in response to various stimuli [59]. The expression of NR4A1 is under nutritional control and is involved in glucose homeostasis in several tissues and in modulating mitochondrial function [60–62]. Our GSEA results contribute to the idea of altered mitochondrial metabolism in both the TN and the BNST. Our behavioural results suggest differences in energy expenditure, given that Isolated animals were overall less active than Paired animals, which could also explain the potential neural differences in metabolism. Isolation-induced changes in metabolism are important to consider in many studies, particularly those involving isolation for respirometry.

In addition to downregulation of *NR4A1*, another three immediate early response genes (*FOS*, *ARC*, *EGR1*) were downregulated in the BNST. This is similar to findings in the auditory lobule (a distinct brain region) of female zebra finches isolated overnight [47]. Expression of these genes is rapidly upregulated after neuronal activation [63, 64] and, therefore, the difference in expression between isolated and paired animals could suggest differences in neural activation of this brain region. Both sexual and other social interactions have been shown to elicit activation of the BNST (reviewed in [30]), so it is therefore possible that the higher expression of immediate early genes in this brain region in Paired animals is due to interactions with a female.

The opioid receptor-like 1 (*OPRL1*) gene was upregulated in the BNST after isolation. This gene encodes the nociceptin opioid peptide receptor (NOP), which is activated by the nociceptin/orphanin FQ (N/OFQ) peptide. At the level of the brain, the N/OFQ–NOP system is involved in pain perception, feeding, anxiety, locomotion, and response to stress [reviewed in [65]). Some of the effects of N/OFQ are complex and site-specific and still being clarified. For example, when administered supraspinally, N/OFQ leads to increased pain perception (hyperalgesia), but spinal N/OFQ is antinociceptive [65]. In the BNST, local administration of N/OFQ can block CRF-induced anorexia and CRF-induced anxiety (reviewed in [66]). The upregulation of NOP in the BNST could therefore lead to altered pain perception, anxiety, and food intake, all of which are responses important in many behavioural tests. The GSEA results suggest changes to glutamatergic synapse in the BNST. Glutamatergic transmission in this region is important in modulating stress and anxiety [67–69], which further contributes to the idea that social isolation may have activated anxiogenic responses.

In mammals, it is well established that modulation of neural circuits of the amygdala by the serotoninergic system is involved in fear-related behaviours [70]. Lesion studies of the arcopallium/amygdala complex (a brain region encompassing the TN) have also linked this region to fear- and escape-behaviour in birds [71, 72]. Serotonin/serotonin transporter systems also appear to modulate fear-related behaviours in chickens [73, 74]. In the TN, the serotoninergic system was shown to be involved in the modulation of ingestive behaviours in pigeons [75]. The increase in the expression of the gene encoding the 5-HTR1E serotonin receptor in the TN could suggest changes to serotonin activity in this region, with consequences for fear and ingestive behaviours. The functions of this particular serotonin receptor are not as well-known as those of other serotonin receptors, but its activation appears important for promoting neuronal survival against oxidative stress [76], which our results suggest was increased in the TN and BNST.

Although we did not find significant differences in circulating corticosterone between treatments, it is possible that differences occurred earlier and influenced the neural results found. In addition, it is important to note that the corticosterone analysis was done on a limited set of samples where we collected sufficient plasma from. While one study in zebra finches isolated for two days did not find differences in corticosterone between group-housed and isolated finches [77], a separate study found an elevation of corticosterone at 10 min (but not 30 min) after isolation from a mate [52]. In human cells, a genome-wide analysis identified 209 genes that show significant changes in expression in response to glucocorticoids [78]. Two genes that differed significantly in expression between treatments in our study coincide with genes that were downregulated by treatment with glucocorticoids on that human study: *NR4A1* and *MIDN*. These genes showed lower expression in Isolated animals relative to Paired animals. It is therefore possible that some neural responses observed could be influenced by early differences in corticosterone between the treatments, but changes in corticosterone are not necessary for social isolation to lead to neural changes (e.g., [47, 79]).

The effects of social isolation and the duration threshold at which animals physiologically switch from experiencing acute to chronic effects of social isolation likely depend on various factors, including the social system and lifespan of the target species, seasonality, the environment in which social isolation takes place (e.g., novel or familiar), and the causes of the isolation (e.g., social rejection or defeat versus loss of social partner due to death). Regardless of these factors, the reality is that in many laboratory tests, animals are separated from their group or cagemate for short periods of time. Therefore, studying the effects of short-term social isolation is important for the interpretation of experimental results where brief social isolation is necessary. Here, we found that an isolation period of 3 h led to coordinated changes in transcripts associated with synaptic signalling and transmission and with metabolism. It is important to note that our study included only males. It is highly likely that sex differences exist in the neural response to social isolation. Studying these sex differences will be critical for determining when the effect of sex derives from a larger impact of brief social isolation in one of the sexes, rather than some other aspect of interest in the experimental manipulation. For example, a screen of behavioural experiments assessing neuropsychiatric phenotypes in mice [80], many of which involve placing animals alone in test arenas or in cages, found that female C57BL/6 N mice were less anxious than males because they spent more time in the centre of the arena in the open field test. Are females of that strain overall less anxious than males, or do they respond to social isolation differently? In addition to obtaining new knowledge on the effects of social isolation, developing tests that avoid the need for isolating animals will help circumvent this confounding variable. The IntelliCage allows for the assessment of a variety of behaviours and cognitive function of group-housed rodents [81]. Use of sophisticated RFID and detection systems has also allowed researchers to study spontaneous cage activity and wheel running in group-housed rodents [82], as well as time spent in groups, group size, territory size, and drinking behaviour, in groups of hundreds of mice living in a natural environment [83, 84]. In addition, automated image-based tracking is continuously and rapidly evolving [85], now permitting for tracking and pose-estimation of groups of animals (e.g., [86]). In conclusion, when social isolation cannot be avoided during an experiment by use of more sophisticated methods, its effects need to be understood to contextualize the experimental results found.

### Electronic supplementary material

Below is the link to the electronic supplementary material.

Supplementary Material 1

## Data Availability

Sequencing statistics, differential expression results, GSEA results, and raw behavioural and corticosterone data are provided in the supplementary tables. RNA-sequencing datasets are available from the Gene Expression Omnibus database (accession #GSE203528).
